# Characterization of physiological and antioxidant responses in *Run1Ren1 Vitis vinifera* plants during *Erysiphe necator* attack

**DOI:** 10.3389/fpls.2022.964732

**Published:** 2022-10-06

**Authors:** Viviana Sosa-Zuniga, Vera Martínez-Barradas, Carmen Espinoza, Ricardo Tighe-Neira, Álvaro Vidal Valenzuela, Claudio Inostroza-Blancheteau, Patricio Arce-Johnson

**Affiliations:** ^1^ Departamento de Genética Molecular y Microbiología, Pontificia Universidad Católica de Chile, Santiago, Chile; ^2^ Facultad de Agronomía e Ingeniería Forestal, Pontificia Universidad Católica de Chile, Santiago, Chile; ^3^ Facultad de Ciencias de la Salud, Instituto de Ciencias Biomédicas, Universidad Autónoma de Chile, Santiago, Chile; ^4^ Departamento de Ciencias Agropecuarias y Acuícolas, Facultad de Recursos Naturales, Universidad Católica de Temuco, Temuco, Chile; ^5^ Research and Innovation Centre. Biotechnology vegetal Unit, Foundation Edmund Mach, San Michele all'Adige, (TN), Italy; ^6^ Núcleo de Investigación en Producción Alimentaria, Facultad de Recursos Naturales, Universidad Católica de Temuco, Temuco, Chile; ^7^ Agrijohnson Ltda., Plant Biotechnology Department, Miraflores, Curacavií, Chile

**Keywords:** powdery mildew, *Vitis vinifera*, *Run1Ren1*, resistance genes, photosynthesis, oxidative stress, *Erysiphe necator*, plant pathogens

## Abstract

Grapevine is a fruit crop of major significance worldwide. Fungal attacks are one of the most relevant factors affecting grapevine yield and fruit quality, and powdery mildew caused by *Erysiphe necator* is one of the most harmful fungal diseases for this fruit-bearing species. Incorporating resistance genes such as *Run1* and *Ren1* in new vine selections offers a sustainable alternative to control the disease. These combined loci produce an immune response that prevents the development of the disease. However, to date studies are lacking concerning whether this response generates alterations in the physiological and antioxidant parameters of resistant plants in the presence of the fungus or if it has an associated energy cost. Therefore, the main goal of our research was to determine if *Run1Ren1* plants present alterations in their physiological and biochemical parameters in the presence of the fungus. To achieve this target, a previously characterized resistant *Run1Ren1* genotype and the susceptible Carménère cultivar were analyzed. We evaluated photochemical parameters (Fv’/Fm’, ΦPSII and ETR), net photosynthesis (*Pn*), photosynthetic pigments, transpiration (*E*), stomatal conductance (*g_s_*), oxidative stress parameters (MDA), antioxidant activity, and phenols. Our results show that the physiological parameters of *Run1Ren1* plants were not negatively affected by the fungus at 10 days post-inoculation, contrasting with alterations observed in the susceptible plants. Therefore, we propose that the resistance response triggered by *Run1Ren1* is physiologically and biochemically advantageous to grapevines by preventing the development of powdery mildew infection.

## Introduction

Grapevine (*Vitis vinifera*) is one of the most important fruit crops worldwide (OIV, 2021), with a total planted area of 7.3 million hectares. Since grapevines are cultivated globally, plants are exposed to a wide range of environmental conditions, including changes in temperature, soil composition, humidity, water availability, sunlight, and the presence of pathogens. In particular, biotic agents are one of the most relevant and widely distributed agricultural challenges, causing substantial losses in fruit yield (Armijo et al., 2016), either during the pre-harvest or post-harvest phase. Powdery mildew (PM; *Erysiphe necator)* is the most critical pre-harvest disease for grapevine due to its highly destructive force and its presence worldwide. This pathogen decreases cluster weight and affects fruit ripening (Pool et al., 1984; Wicks et al., 1985; Reuveni and Reuveni, 1995). In physiological terms, it lowers photosynthetic and transpiration rates, and stomatal conductance (Pool et al., 1984; Moriondo et al., 2005). To control the cellular machinery and survive, *E. necator* reprograms the metabolism of the host cells (Pimentel et al., 2021), which reduces the abundance and availability of metabolites from glycolysis and photorespiration, as well as photosynthetic proteins (Marsh et al., 2010). Most studies have determined how PM affects grapevines at the molecular level. Weng et al. (2014) made a transcriptomic analysis of *V. pseudoreticulata*, a resistant wild grape, under inoculated conditions. They observed an up-regulation of genes related to plant-pathogen interaction; salicylic acid (SA) and jasmonic acid (JA) response pathway; systemic acquired resistance (SAR); hypersensitive response (HR); and flavonoid biosynthesis under inoculated conditions. On the contrary, genes associated with cell replication and methylation were down-regulated.

On the other hand, the physiological and antioxidant responses of the host have received relatively little attention. Although it is known that gas exchange is affected by PM in grapevines (Pool et al., 1984), little is known about the behavior of photochemical parameters, and how infection alters plant performance. Currently, there are few studies on the responses during fungus infection of maximum quantum yield (Fv’/Fm’), effective quantum yield (ΦPSII), electron transport rate (ETR), and the content of associated pigments (chlorophyll and carotenoids) (Yin et al., 2021). Concerning these pigments, Moriondo et al. (2005) described that the concentration of chlorophyll*-a* and *-b* decreased in grapevine leaves infected with PM. Although little research has been performed on vines regarding the physiological effects generated by this fungus on the host, evidence from other woody species does exist. For instance, Glynn and Fraser (2002) studied the physiological response of English oak (*Quercus robur*), hedgehog rose (*Rosa ‘*rugosa’), and horse chestnut (*Aesculus hippocastanum*) to infection by their compatible PM species: *Sphaerotheca pannosa* var. *rosa*, *Phyllactinia* sp. and *Erysiphe flexuosa*, respectively. In all of these woody species, photosynthesis rate, ETR, chlorophyll, and carotenoid content declined in infected leaves. In the case of infected herbaceous plants, photochemical parameters have aslo been examined. For example, Brugger et al. (2017) described that ΦPSII and chlorophyll content fell during the infection of barley (*Hordeum vulgare*) with cereal PM (*Blumeria graminis*).

Plants generate reactive oxygen species (ROS) in their primary metabolism. These are not harmful under normal conditions since cells have evolved mechanisms to neutralize them quickly. However, when the plant is under stressful situations, such as pathogen attack, an imbalance of ROS is formed, generating oxidative stress (Czarnocka and Karpiníski, 2018). An indicator of this disequilibrium is lipid peroxidation, which produces malondialdehyde (MDA) from polyunsaturated fatty acids (Alcheí, 2019). MDA is thus as an indicator of the oxidative state of the plant, and is associated with the presence of stress conditions and cellular damage (Aly et al., 2012; Lanubile et al., 2015). Among the main mechanisms for ROS detoxification is the ascorbate-glutathione pathway (Foyer and Noctor, 2011), in which dehydroascorbate reductase (DHAR) plays a crucial role in the antioxidative cell system through the reduction of dehydroascorbate (DHA) to ascorbate (AsA) (Foyer and Noctor, 2011). For that reason, the abundance of DHA and AsA is used as an indicator of oxidative stress. Other common measurements to indirectly evaluate the oxidative status of the tissue are antioxidant activity and phenol concentration, both of which increase under stress conditions. For example, in flax (*Linum usitatissimum* L.) during PM (*Oidium lini Skoric*) infection, there is a negative correlation between phenols and AsA with PM infection severity (Aly et al., 2012). On the other hand, MDA positively correlates with PM severity, concordant that a more severe disease generates more cellular damage in the host.

Nowadays, control of *E. necator* is mainly achieved by intensive fungicide applications that are associated with a high cost to the growers, negative environmental impact, and human health consequences (Belpoggi et al., 2006; Calviello et al., 2006; Rossi et al., 2006; Cecconi et al., 2007; Garciía-Garciía et al., 2016). *E. necator* can adapt and evolve on a short spatial and temporal scale. Its tolerance to temperature variations has allowed it to grow in a wide range of climatic conditions (Bois et al., 2017). This fungus is classified into two genetic groups, A and B, which differ in the moment of the season when they attack, the severity of the infection, and their genetic variability (Montarry et al., 2009). It has also been reported that pathogen sexual reproduction is favored under slight rises in temperature (Legler et al., 2012). This attribute can give an advantage to PM under the climate change scenario, which generates an increase in mean temperatures in most land and ocean regions and changes in precipitation.

Agricultural production, particularly that of wineries is looking to decrease fungicide applications. Therefore, there is a need to develop fungi-resistant genotypes to fulfill the needs of consumers and producers (Delrot et al., 2020). Several PM-resistant genes and loci, such as the *Run* and *Ren* gene family have been studied to achieve this goal. Among them, the *Run1* gene and *Ren1* locus stand out due to their synergic effect, which generates a strong defense response (Agurto et al., 2017) that produces complete resistance (Hoffmann et al., 2008; Feechan et al., 2013; Agurto et al., 2017). Nevertheless, there is a need to examine whether the presence of these loci in the grapevine genome affects plant physiology following PM attack. For that reason, in this article we evaluate the physiological performance of resistant *Run1Ren1* grapevine plants and susceptible Carménère plants after exposure to PM.

## Materials and methods

### Plant material

Plants of a resistant genotype (P09-105-59) and a susceptible cultivar (Carménère) were used. The resistant genotype was previously characterized by Agurto et al. (2017) who demonstrated the presence of the *Run1* (GenBank accession number: JQ904636.1) and *Ren1* loci in its genome. The Carménère cultivar (here on referred to as susceptible) was used as a susceptible control that does not harbor either the *Run1* or the *Ren*1 locus. All plants were grown as cuttings in pots containing peat and perlite in a 1:2 ratio, in greenhouses located in the Curacaví experimental field, Chile (33° 24’01.0’’ S 71° 03’ 17.6’’ W). The greenhouse was maintained at 24 ± 2°C, with a 16h photoperiod, a relative humidity of 35-40% and a light intensity of 150 μmol m^-2^ s^-1^.

### Phenotype and genotype characterization of plant material

The phenotype of the plant material was evaluated by inoculating five leaf discs of each plant with *E. necator*. The leaves used for the experiment were selected according to the criterion of being leaves of similar size and age from the upper third of the plant. The use of leaf discs was preferred over using whole leaves because this allowed for heightened standardization of the samples. Leaf discs were created with a cork borer (1 cm diameter). Inoculation was carried out by gentle contact of the abaxial side of leaf discs with infected tissue from other grapevines that had visible spores; the latter did not belong to the experiment itself. Leaf discs were maintained in Petri dishes in a growth chamber at 26 ± 2°C in a 12 hour light and 12 hour dark photoperiod. Ten days post-inoculation (dpi), the presence of PM infection on leaf discs was assessed by visual inspection and then corroborated with a magnifying glass. The leaf samples used for the phenotypic analysis were not used for the genotypic analysis, since the use of fresh tissue was preferred to perform DNA extractions.

Subsequently, new leaf tissue samples were processed to perform the genotypic analysis. Each sample consisted of 100 mg of one leaf that was selected randomly from healthy, fully expanded leaves of each plant. In order to determine the presence or absence of *Run1* and *Ren1* loci in the plants, DNA was extracted from each sample following the protocol of Gambino et al. (2008) with modifications (incubation of 1 hour at 65°C at step two, centrifuge at full speed for 30 minutes at step seven and resuspension in 30 μL sterile distilled water at step ten). Then, a genotype screening was carried out using the Simple Sequence Repeats (SSR) molecular markers VMC8g9 (Barker et al., 2005) and Sc47_20 (Coleman et al., 2009). To prepare the master mix, the following amounts of each component were used: 11.8 μL H_2_O; 4 μL 5X Phusion™ High-Fidelity Buffer (ThermoFisher™, MA, USA); 0.4 μL 10 mM dNTPs (Invitrogen™, MA, USA); 1 μL forward primer (Integrated DNA Technologies^©^, WI, USA), 1 μL reverse primer (Integrated DNA Technologies); 1 μL DNA; 0.6 μL DMSO (ThermoFisher) and 0.2 μL Phusion™ High-Fidelity DNA polymerase (ThermoFisher). The PCR program used was 35 cycles of 54°C for 10s at annealing and 72°C for 30s at extension. A 3% agarose (Sigma-Aldrich SA, MA, USA) gel and GelRed^®^ (Biotium, CA, USA) were used to separate and visualize the amplified fragments.

### Physiological experiment design

For physiological analysis, eight plants from each genotype were inoculated (herein referred to as inoculated plants), and randomly distributed in one greenhouse (GH1). As a control, another group of eight plants of each genotype were separated in another greenhouse (GH2) (herein referred to as non-inoculated plants). Plants were randomly distributed in each greenhouse (**Supplementary Figure 1**). All leaves of GH1 plants were inoculated ten days before measurements, following the inoculation procedure described above. To avoid the involuntary contamination of fungus-free plants, the non-inoculated plants were treated with Captan 12 WP fungicide (ANASAC, Santiago, Chile) seven days before the measurements.

### Fluorescence chlorophyll-a and gas-exchange analysis

The chlorophyll fluorescence parameters were measured in intact fully expanded leaves selected randomly in the middle third of the plants in light conditions using the fluorescence chamber of the IRGA LI-6400XT Portable Photosynthesis System (Li-Cor, Nebraska, USA). Maximum quantum yield [Fv’/Fm’]=(Fmí-F0’)/Fm’ was determined according to Logan et al. (2007), whilst the effective quantum yield of photosystem II [ΦPSII=(Fm’-Fs)/Fm’)’ and electron transport rate [ETR= ΦPSII*α*β*PPFD) were calculated according to Genty et al. (1989). Net photosynthesis ratio (*Pn*, μmol CO_2_ m^-2^s^-1^), transpiration rate (*E*, mmol H_2_O m^-2^s^-1^), and stomatal conductance (*g*s, mol H_2_O m^-2^s^-1^) were also quantified. The measurements were undertaken at 400 ppm CO_2_ and a photosynthetic active radiation of 700 μmol photons m^-2^s^-1^ in the 2021-2022 season, between 9:00 and 12:00 h.

### Determination of photosynthetic pigments

Chlorophyll-*a+b*, Chl-*a*+*b* (graphed as total chlorophyll), the Chl-*a/b* ratio, and carotenoids were extracted according to Medeiros et al. (2017), with modifications. Approximately 15 mg of fresh material was ground in liquid nitrogen and 0.7 mL pure methanol. The samples were shaken vigorously at 80°C for 20 min. Afterward, they were centrifuged at 17000 g at 4°C for 10 min, and chlorophylls were calculated according to Porra et al. (1989). Chlorophyll-*a* and *–b*, and carotenoids were measured at 653, 666, and 470 nm, respectively with a spectrophotometer (microplate reader EPOCH, Biotek, Winooski, VT, USA) and expressed in mg g^-1^FW.

### Lipid peroxidation assay

Malondialdehyde (MDA) was determined by the modified method described by Du and Bramlage (1992) using thiobarbituric acid reacting substances (TBARS). Approximately 0.15 g of freshly ground leaves were used for analysis. Absorbance was measured at 440, 530, and 660 nm in a UV/VIS spectrophotometer (UNICOR 2800, New Jersey, USA). Results were expressed as MDA content (nmol MDA g^-1.^FW), a secondary product of the oxidation of polyunsaturated fatty acids (Hodges et al., 1999).

### Antioxidant and phenol assays

Leaf antioxidant activity (AA) was determined based on the method described by Chinnici et al. (2004) using the 2.2-diphenyl-1-picrylhydrazyl (DPPH) free radical scavenging assay. Leaf samples were ground in liquid nitrogen and soaked in 1 mL 80:20 (v/v) methanol: water. Absorbance was measured at 515 nm using Trolox as the standard and a UV/VIS spectrophotometer (UNICOR 2800). The values were expressed in mg Trolox equivalents g^-1^FW. The total phenols (TP) were determined by the Folin-Ciocalteu method, as described by Slinkard and Singleton (1977). Absorbance was measured at 765 nm and expressed in mg chlorogenic acid equivalents (CAE) g^-1^ FW.

### Ascorbic acid determination

Ascorbic acid content (ASC) was quantified by the protocol described by Kampfenkel et al. (1995) with minor modifications. About 50 mg of leaf material were ground in liquid nitrogen, homogenized in 330 μL 1 mM EDTA + 0.1 M HCl, and centrifuged at 12000 g at 4°C for 10 min. An aliquot of 20 μL was used to measure the absorbance at 520 nm in a microplate spectrophotometer (EPOCH). Ascorbic acid (AsA) levels were determined using sodium ascorbate as standard from a standard curve. The content of dehydroascorbate (DHA) was calculated by subtracting the measurements without N-ethylmaleimide (NEM). Results of ASC were expressed as mmol g^-1^FW, and the AsA/DHA ratio was also determined.

### Statistical analysis

A completely randomized experimental design was used. The experiment was composed by two factors (two genotypes * two infection conditions), three repetitions were measured in each experimental unit, composed of one mature leaf of each plant. For all the statistical analysis RStudio with R.4.2 was used: for the two ways ANOVA with p-value ≤0,05 we used, followed by Tukey’s HSD posthoc test with 95%confidence level. Also, we carried out Principal Components Analysis (PCA), using RStudio R.4.2 software.

## Results

### Phenotype and genotype analysis of segregant lines

**Figure 1:**
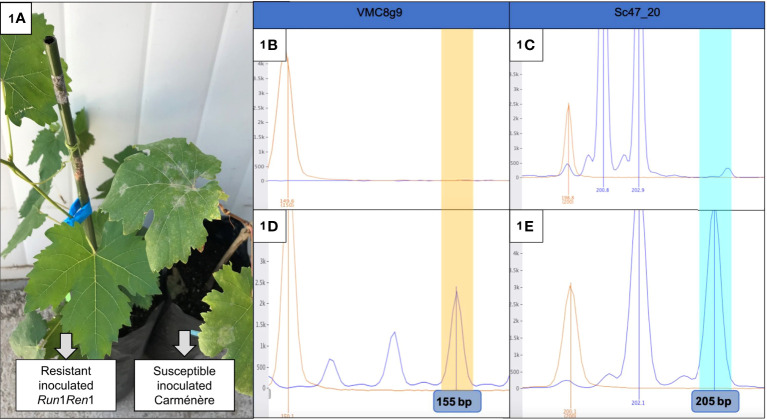
Phenotypic and genotypic analysis of P09-105-59 and Carménère plants. **(A)** Photo of the resistant Run1Ren1 P09-105-59 (left) and a susceptible Carméńère (right) inoculated plants. The first plant did not show any visible symptoms, unlike the second plant, that had a perceptible presence of fungus structures (white/grey dust); **(B–E)** Electrophoretogram of SSR analysis of resistant and susceptible Carméńère plants. VMC8g is linked to *Run1* and Sc47_20 is linked to *Ren1*. P09-105-59 resistant genotype carried Run1 **(D)** and *Ren1* **(E)**, on the other hand, Carméńère did not have any of them **(B, C)**. Polymorphic fragments for VMC8g9 (155 bp) and Sc47_20 SSR (205 bp) are highlighted in yellow and blue, respectively.

We were interested in the physiological response of *E. necator* infected grapevine plants, in the absence and presence of the *Run1Ren1* resistant genes. Therefore, to begin the study it was first necessary to characterize the plants both phenotypically and genotypically. To do so, leaf discs were inoculated with *E. necator*, and infection symptoms monitored. In the phenotype analysis, leaf discs of resistant plants did not show any visible symptom of PM attack at 10 dpi. On the other hand, Carménère leaf disks displayed the first visible signs (white-gray dust on leaves) at 7 dpi, and all discs showed symptoms at 10 dpi. The same results were also seen at the whole-plant level **(** **Figure 1A** **)**. Since the resistance provided by *Run1* and *Ren1* generates a total absence of symptoms (Agurto et al., 2017), the lack of symptoms was an expected outcome of this experiment. Subsequently, the presence of the *Run1* and *Ren1* loci was evaluated in a genotypic SSR analysis to corroborate the phenotype results. In the case of plants with a susceptible phenotype, SSR detected the lack of *Run*1 and *Ren*1 loci (**Figures 1B, C** **)**, whilst the presence of *Run1* and *Ren1* loci was associated exclusively with PM-resistant grapevines (**Figures 1D, E**). These results showed that the presence of *Run1* and *Ren1* confers resistance to *E. necator* infection, as previously described (Agurto et al., 2017).

### Photochemical parameters

In our research, we did not observe significant differences in Fv’/Fm’ between susceptible and resistant plants, or between non-inoculated and inoculated groups **(** **Table 1** **)**. For ΦPSII, a substantial increase of over 40% was observed in inoculated susceptible and resistant groups compared to non-inoculated groups of the same cultivar or genotype **(** **Table 1** **)**. For ETR, no significant differences were observed between any of the groups studied (**Table 1**).

**Table 1:** Summary of the parameters associated with chlorophyll fluorescence and gas exchange. In the case of chlorophyll fluorescence, maximum quantum yield (Fv’/Fm’), the effective quantum yield PSII (ΦPSII), and electron transport rate (ETR) were evaluated.

VARIABLES	RESISTANT		SUSCEPTIBLE	
Non-Inoculated	Inoculated	Non-Inoculated	Inoculated
Maximum Quantum Yield (Fv’/Fm’)	0.53 ± 0.032 a	0.51 ± 0.056 a	0.53 ± 0.037 a	0.52 ± 0.049 a
Effective Quantum Yield PSII (ΦPSII)	0.04 ± 0.020 c	0.07 ± 0.017 ab	0.04 ± 0.010 c	0.06 ± 0.014 b
Electron Transport Rate (ETR)	22.39 ± 12.457 a	25.47 ± 6.169 a	17.69 ± 5.170 a	22.1 ± 2.755 a
Net Photosynthesis (*Pn*, μmol Co_2_ m^-2^s^-1^)	4.89 ± 0.770 a	4.49 ± 0.686 ab	4.75 ± 0.779 a	3.74 ± 0.292 b
Stomatal Conductance (*g_s_*, mol H_2_O m⁻² s⁻¹)	0.05 ± 0.013 a	0.04 ± 0.020 a	0.04 ± 0.006 a	0.04 ± 0.009 a
Transpiration (*E*, mmol H_2_O m⁻² s⁻¹)	1.32 ± 0.345 a	1.37 ± 0.533 a	1.23 ± 0.155 a	1.13 ± 0.236 a

Within the gas exchange parameters, net photosynthesis (μmol CO_2_ m^-2^s^-1^) (*Pn)*, stomatal conductance (mol H_2_O m^−2^ s^−1^) (*g_s_*), and transpiration (mmol H_2_O m^−2^ s^−1^) (*E*) were measured. Lowercase letters indicate statistically-significant differences between inoculated (with *E. necator*) and non-inoculated in factors interaction (genotypes*infection condition). A two-way ANOVA test and the Tukey multiple comparison *post-hoc* test (*p* ≤ 0.05) (n=8 ± SD) were performed.

### Gas exchange parameters

In the non-inoculated group, basal *Pn* rate was unchanged between the susceptible cultivar and the resistant genotype **(** **Table 1** **)**. In the case of inoculated groups, the same trend was seen, in that there was no significant difference between the inoculated groups of susceptible and resistant plants. However, the magnitude of the decrease in the value of *Pn* between the inoculated and non-inoculated groups of resistant and susceptible plants was different. The group of inoculated resistant plants did not show a significant decrease (by 8.2%) compared to the same genotype of the non-inoculated group. On the other hand, in inoculated susceptible plants, the reduction in their photosynthesis was significant (by 21.3%) with respect to the same cultivar of non-infected plants. For *gs* and *E*, no significant differences were observed in any of the groups studied **(** **Table 1** **)**.

### Photosynthetic pigments

**Figure 2:**
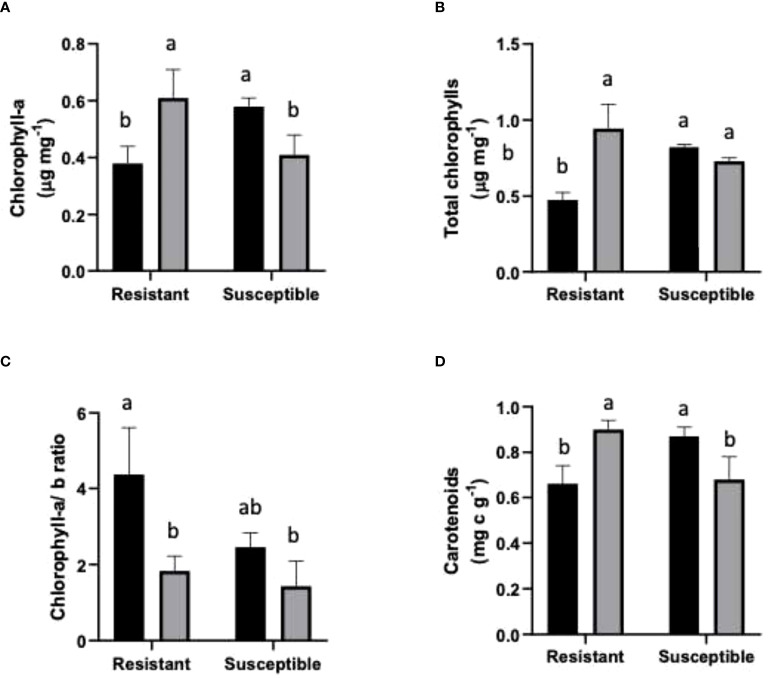
Photosynthetic pigments in resistant and susceptible plants. **(A)** Chl-*a* μg mg^-1^ FW; **(B)** Chl-*a+b* µg mg^-1^ FW; **(C)** Chl- *a/b* ratio; and **(D)** Carotenoids (mg g^-1^ FW). Black bars represent non inoculated plants, and grey bars indicate *E. necator* inoculated plants. Lowercase letters indicate statistically significant differences among inoculated and non-inoculated in factors interaction (genotypes*infection condition). Two-way ANOVA test and the Tukey multiple comparisons *post-hoc* test (*p ≤* 0.05) (n=3 ± SD).

In the case of Chl*-a* content, non-infected susceptible plants had an 18% higher content than non-inoculated resistant plants. Inoculated susceptible plants had a lower Chl*-a* content compared to the non-inoculated samples of the same cultivar. On the other hand, inoculated resistant plants showed a significant increase in Chl-*a* content with respect to non-inoculated resistant plants (**Figure 2A**). Total chlorophylls significantly increased in resistant inoculated plants, while non-significant changes were observed in inoculated susceptible plants **(** **Figure 2B** **)**. The Chl-*a/b* ratio was reduced in inoculated plants compared to non-inoculated plants of both genotypes **(** **Figure 2C** **)**. In inoculated resistant and susceptible plants, the Chl-*a/b* ratio decreased by 58% and 41.7%, respectively.

In the case of carotenoids, the resistant non-inoculated plants had a 24% lower content compared to resistant inoculated plants. However, in the susceptible plants, a contrasting behavior was observed; susceptible inoculated plants had 21% fewer carotenoids than non-infected susceptible plants (**Figure 2D**).

### Lipid peroxidation, total phenols, and antioxidant activity

**Figure 3:**
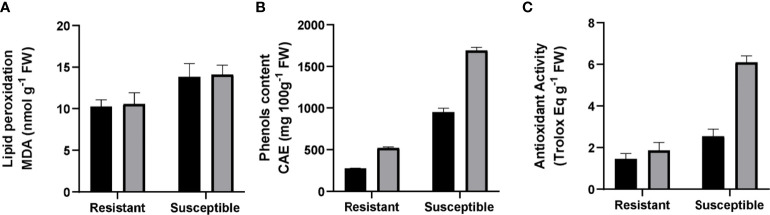
Evaluation of antioxidant capacity in resistant and susceptible plants. **(A)** Malondialdehyde (nmol g^-1^ FW). **(B)** Phenols, expressed in chlorogenic acid equivalents (CAE µg g^-1^ FW). **(C)** Antioxidant activity, measured by a 2,2-diphenyl-1-picrylhydrazyl radical scavenging assay (DPPH) (Trolox Eq g^-1^ FW). Black bars represent non inoculated plants, and grey bars indicate *E. necator* inoculated plants. Lowercase letters indicate statistically significant differences among inoculated and non-inoculated in factors interaction (genotypes*infection condition). A two-way ANOVA test and the Tukey multiple comparison *post-hoc* test (*p* ≤ 0.05) (n=3 ± SD) were performed.

Our experiment showed a significant difference in the baseline of lipid peroxidation levels between the genotypes. Resistant plants had 30% less MDA concentration than susceptible plants. There were no differences between non-infected and infected plants in either genotype (**Figure 3A**). Another parameter evaluated was the phenolic compound concentration. An increase in phenol production has been associated with greater stress tolerance (Dixon and Paiva, 1995) due to their scavenging action of ROS. Our analyses showed a 6.6-fold higher level of phenols in non-inoculated susceptible plants compared to non-inoculated resistant ones. There was an increase in phenolic compounds in inoculated groups compared to their non-inoculated counterparts. Susceptible grapevine had 77.6% more total phenols, and in resistant plants the increase was 88.8% (**Figure 3B**). Regarding antioxidant activity, this was 30% lower in non-inoculated resistant plants than in susceptible plants with the same treatment. For the inoculated group, resistant plants suffered no changes in antioxidant activity, while the susceptible plants presented an increase of 101% in the same parameter (**Figure 3C**).

### Ascorbic acid

**Figure 4:**
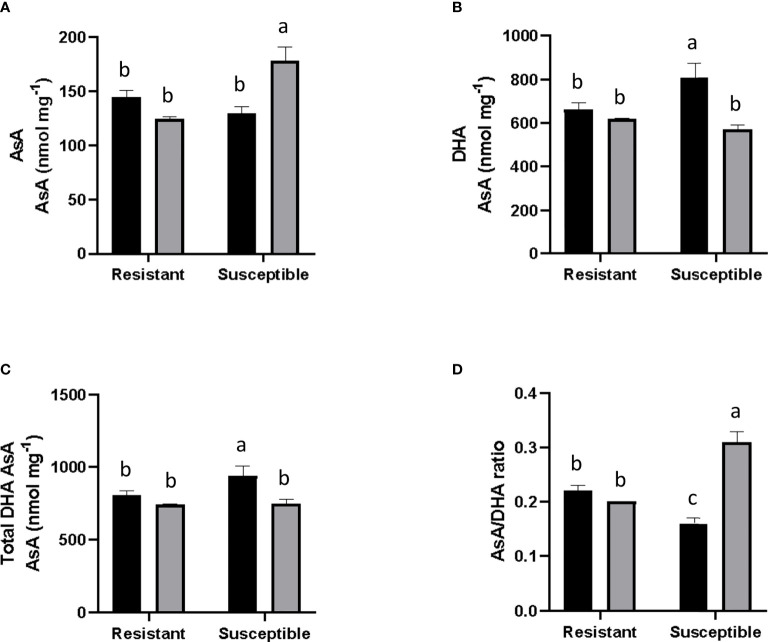
Evaluation of Ascorbic Acid content and associated parameters. Gray and black bars correspond to *E*. *necator* inoculated and non-inoculated plants. **(A)** Ascorbic Acid (AsA) levels; **(B)** Dehydroascorbic Acid (DHA); **(C)** Total AsA and DHA levels; and **(D)** Asa/DHA ratio. Black bars represent non inoculated plants, and grey bars indicate *E*. *necator* inoculated plants. Lowercase letters indicate statistically significant differences among inoculated and non-inoculated in factors interaction (genotypes*infection condition). A two-way ANOVA and the Tukey multiple comparison *post hoc* test (*p* ≤ 0.05) (n=3 ± SD) were performed.

An increase of 18.1% was observed in the amount of AsA in inoculated susceptible grapevine. In contrast, the inoculated resistant plants decreased their AsA content to a similar level seen in non-inoculated susceptible plants (**Figure 4A**). For DHA, in non-inoculated plants, a 5% lower amount of basal DHA content was observed in the resistant genotype compared to the susceptible one. In both inoculated groups, DHA levels were reduced; the susceptible group showed a notorious decrease of 23%, whilst levels fell marginally in the resistant group (by 0.9%, **Figure 4B**). For total DHA-AsA content, a 1.7% higher basal level was measured in non-inoculated susceptible plants compared to resistant ones. Also, in both groups, total DHA-AsA levels decreased after PM inoculation. Specifically, DHA-AsA levels decreased in inoculated plants compared to the corresponding non-inoculated genotype, a fall which was significant in the case of the inoculated susceptible group. On the other hand, this parameter was 3.1% lower in the inoculated resistant group, a reduction which was not statistically-significant (**Figure 4C**). Finally, it was observed that the AsA/DHA ratio was 17% higher in resistant plants than in susceptible ones under non-inoculated conditions. In an inoculated state, in resistant plants, the AsA/DHA ratio remained constant, while in susceptible plants it rose by 70% (**Figure 4D**).

### Principal components analysis

**Figure 5:**
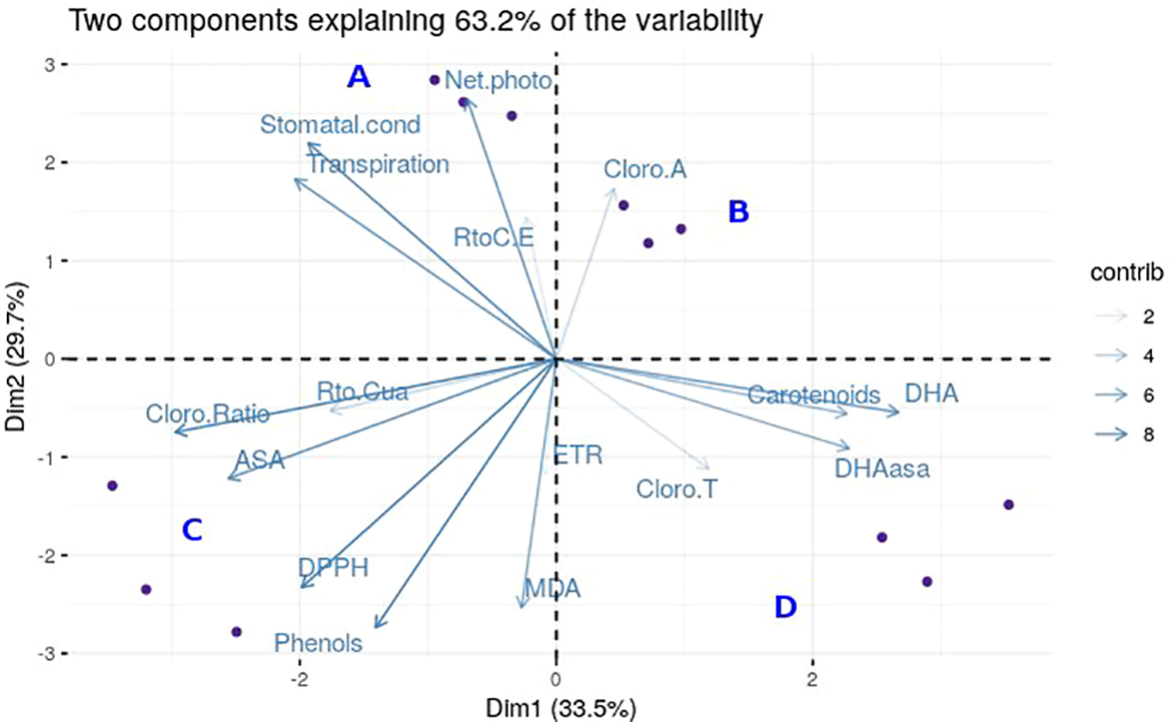
Graphical representation of Principal Components Analysis. For the PCA analysis, blue dots represent each individual. The different genotypes with the two different treatments are shown in each cuadrant: Resistant Non-Inoculated **(A)**, Resistant Inoculated **(B)**, Susceptible Inoculated **(C)**, and Susceptible Non-Inoculated **(D)**. Each blue arrow represents the vector of each variable with the following names: “Cloro. Ratio” for Chl-*a/b*, “Transpiration”, “Stomatal.cond” for stomatal conductance, “RtoC.E” for effective quantum yield, “Net.photo” for net photosynthesis, “Cloro.A” for Chl-*a*, “Carotenoids”, “DHA”, “DHAasa” for DHA.AsA, “CloroT” for total chlorophylls, “MDA” for lipid peroxidation, “ETR” for electron transport rate, “Phenols”, “DPPH” for antioxidants, “ASA”, and “Rto.Cua” for maximum quantum yield. The transparency of the vectors is related to the contribution (contrib) of each one.

By carrying out a PCA, we obtained a summary of the relevant variables for the separation of the four groups of plants analyzed, considering the 17 variables that were measured. We obtained a complete individualization of the four groups by PC1 and PC2, accounting for 85.2% of the variability (**Figure 5**). Analyzing the PCA results, we conclude that five variables do not influence the differences between groups: the total chlorophyll content, ETR, Fv’/Fm’, ΦPSII and chl-*a*. For the differentiation between resistant and susceptible genotypes, the most relevant variables are net photosynthesis, lipid peroxidation, phenols and antioxidant activity.

## Discussion

### Inoculated resistant plants maintained their net photosynthesis rate

Photosynthesis is controlled by biochemical and gas diffusion processes (Yin et al., 2021). To estimate the physiological stage of the studied plants, we evaluated their Fv’/Fm’, ΦPSII, and ETR under non-inoculated and inoculated conditions. These parameters reflect the photochemical component of photosynthesis, through the analysis of the function of photosystem II (PSII) and the elements of the electron transport chain (Kalaji et al., 2016). In our study, the analysis of chlorophyll fluorescence showed that during the different treatments with *E. necator* in resistant and susceptible plants, Fv’/Fm’ did not vary significantly, with a range around 0.53 in the different treatments. A similar behavior was observed in a study with downy and powdery mildew on grapevine leaves, where Fv’/Fm’ did not differ between non-inoculated and inoculated plants (Moriondo et al., 2005). No differences were found in ETR between treatments in our study, although ΦPSII increased significantly (from 0.04 to 0.07) between non-inoculated and inoculated plants, respectively (**Table 1**). This agrees with a study of chlorophyll fluorescence in light conditions where the values fluctuated between 0.67 to 0.77 in untreated and treated gramineous plant species (Savvides and Fotopoulos, 2018). This rise may be due to the activation of the acclimatization mechanism of the plant to maintain the photosynthesis rate under stress conditions (Horton et al., 2008; Murchie and Lawson, 2013).

Subsequently, to study if the presence of the fungus in resistant *Run1Ren1* plants generated an additional energy cost, we measured photosynthesis rate and gas exchange variables, such as *gs* and *E* (Moriondo et al., 2005). This required comparing the results of susceptible and resistant genotypes to determine the basal levels of these parameters in non-inoculated plants, and analyzing whether gas exchange variables showed a differential response in inoculated plants depending on the genotype. In the case of *Pn*, it was found that inoculation generated a slight but non-significant decrease in the resistant genotype and a considerable reduction of this parameter in the inoculated susceptible cultivar (**Table 1**). These results are consistent with previous reports that described a drop in the carbon assimilation rate of susceptible plants during *E. necator* infection (Pool et al., 1984; Shtienberg, 1992; Moriondo et al., 2005). It has been suggested that the fall in photosynthesis is caused by a reduction in the transcription of genes associated with the Calvin and Benson cycle (Fung et al., 2008), which generates a lower abundance of photosynthetic proteins required for fixing CO_2_ ( (Marsh et al., 2010).

### Chl-*a*, Chl-*a/b*, total chlorophyll and carotenoid contents changed in inoculated plants

Chlorophylls and carotenoids play a crucial role in light harvesting; therefore, their levels have been used to indicate plant photosynthetic activity (Kalaji et al., 2016). Chlorophyll-*a* (Chl*-a*) and chlorophyll-*b* (Chl*-b*) are both present in chlorophyll-protein complexes; however their location differs as Chl-*a* is located in the photosynthesis reaction center, whilst Chl-*b* is abundant in light-harvesting complexes (Wang et al., 2014). The relation between chlorophylls was calculated to analyze how biotic stress affected the photosynthetic apparatus. Besides their photosynthetic functions, carotenoids protect plants against photooxidative processes due to their ability to scavenge singlet molecular oxygen and peroxyl radicals (Stahl and Sies, 2003). To our knowledge, there are no previous reports of chlorophyll content measurements in grapevines carrying PM resistance genes, so comparing our results with those from similar studies is not possible. Nevertheless, at the molecular level, a fall in the transcription of the genes required for the synthesis of Chl-*a* tetrapyrroles has been reported (Fung et al., 2008). Our results are in line with such an observation, as a decrease in Chl-*a* content in the susceptible inoculated cultivar (**Figure 2A**) was found, probably due to a decrease in tetrapyrrole synthesis.

On the other hand, the resistant inoculated genotype exhibited the opposite behavior since they harbored greater Chl-*a* levels (**Figure 2A**). In the case of total chlorophylls in susceptible plants, their content remained stable between inoculated and non-inoculated groups (**Figure 2B**). Contrasting with susceptible plants, inoculated resistant plants had a lower level of total chlorophylls than non-inoculated resistant plants (**Figure 2B**). The analysis of the Chl-*a*/*b* ratio in the resistant genotype **(** **Figure 2C** **)** is similar to a previous report of PM infection in other species, as well as signaling a corresponding increase in carotenoid levels in leaves for light energy quenching (**Figure 2D**) (Wang et al., 2014). The findings of Wang et al. (2014) are similar to our results for susceptible plants. In our experiment, inoculated susceptible grapevine had a lower carotenoid level than the corresponding inoculated group, yet in inoculated resistant plants, the infected samples had higher carotenoid levels.

In other species, a decrease in total chlorophyll content in susceptible and resistant plants has been described. Such is the case of barley plants that carry PM (*Blumeria graminis*) resistance genes. Like those evaluated in our experiment, these genes generated a response mediated by programmed cell death (PCD; Brugger et al., 2017). These findings are in contrast to those of our study, suggesting that the overall outcome depends on the host and/or pathogen species being monitored.

### Antioxidant capacity and phenols increased in inoculated susceptible and resistant plants

ROS are a by-product of cell oxidative metabolism. Antioxidant systems maintain ROS homeostasis, a state in which these molecules serve as signaling molecules in different cellular processes. However, this balance can be affected by abiotic or biotic stresses (Czarnocka and Karpiníski, 2018), and imbalance produces lipid peroxidation, which affects membrane fluidity and permeability in cells (Thompson et al., 1987). For that reason, the content of peroxidized lipids is used as an indicator of cellular damage. Particularly in the presence of pathogens, ROS are related to the hypersensitive response, a key mechanism for plant defense. Agurto et al. (2017) associated ROS production with the resistant *Run1Ren1* genotype in grapevines and a later generation of ROS during infection in susceptible cultivars. Our data point towards greater levels of reduced antioxidants in inoculated susceptible plants. These plants have greater antioxidant activity (**Figure 3C**), higher levels of AsA (**Figure 4A**), and a higher phenol concentration (**Figure 3B**). The increase in the reduced state of AsA and the significantly higher antioxidant activity reflect a lower amount of ROS than in inoculated susceptible plants. This relates to previous studies that describe a poor defensive plant response in susceptible *V. vinifera* plants infected by *E. necator* (Cozzi et al., 2013; Agurto et al., 2017). This unaltered cellular environment in susceptible plants allows pathogens to develop due to weak ROS generation in plant tissues, which does not lead to PCD. As an obligate biotrophic fungus, PM needs the infected cells to survive (Viala, 1885). Thus, the absence of PCD allows the infection to continue.

Additionally, we studied the content of AsA, DHA, total DHA-AsA, and the AsA/DHA ratio to evaluate indirectly the oxidation status of the tissue. AsA and DHA, its reduced form, participate in ROS detoxification and prevent cellular damage (Jung et al., 2019). For that reason, the AsA/DHA ratio reflects the degree of adaptation of plants to stress conditions. An imbalance in ROS scavenging processes generates a reduction in AsA/DHA (Anjum et al., 2014). It is also interesting to point out that the AsA pool has been described as a central element in plant defense responses (Foyer et al., 2020). Our results showed that the total pool of AsA by itself in susceptible plants was not enough to stop the fungal infection **(** **Figure 4A** **)**.

In line with these findings is the observation by Hou et al. (2013) that grapevine plants with resistance to *E. necator* have heightened expression of the *VpVTC* gene, an essential gene in AsA synthesis, suggesting that *VpVTC* is part of the regulation of the resistance response. The change in *VpVTC* expression was correlated with increased AsA content in resistant leaves in the presence of the fungus. Nevertheless, our data appear to show another tendency, which we propose is due to temporality. The oxidative burst maximum for resistant *Run1Ren1* plants was reported to occur at 96 hpi (Agurto et al., 2017), and our measurements focus on the performance of the plant in the longer term (10 dpi). It is possible that once resistant plants have overcome the infection due to a short-term HR reaction, the antioxidant systems recovered their redox status by the time our measurements were performed.

### Global association of parameters with resistance obtained by PCA

In the case of resistant *Run1Ren*1 plants, we demonstrated that the *Pn* was maintained after the inoculation with *E. necator*, and levels of lipid peroxidation were lower in resistant plants compared to the susceptible counterparts. In the case of phenols and antioxidants, the resistant plants had greater basal levels of these antioxidants compounds, and in response to PM inoculation (**Figure 5**). Taken together, we propose that these four parameters are key points and markers to differentiate between the susceptibility and resistance to *E. necator* attack in *Run1* and *Ren1* genotypes.

In summary, under the conditions of this study, we observed that *Run1Ren1* resistant plants maintain photosynthetic levels and redox status after *E. necator* inoculation. Therefore, it can be inferred that PM infection does not generate sufficient stress that affects these parameters. Our results show that the *E. necator* fungus did not produce an additional energy cost in resistant *Run1Ren1* grapevines 10 days after inoculation. Additional, longer-term studies are necessary to analyze whether the presence and effects of the resistance loci is associated with variations in yield.

## Data availability statement

The raw data supporting the conclusions of this article will be made available by the authors, without undue reservation.

## Author contributions

Research formulation and conceptualization: VS-Z and PA-J; design and execution: VS-Z, CI-B, and RT-N; samples analysis: CI-B and RT-N; writing-original draft preparation: VS-Z, VM-B, and AV; writing-review and edition: VS-Z, VM-B, CE, CI-B, and PA-J; statistical analysis: VM-B and AV; project supervisor: PA-J; project administration: VS-Z; funding acquisition: VS-Z, AV, and PA-J. All authors read and agree to the published version of the manuscript.

## Funding

VS was supported by the National Research and Development Agency (ANID) of the government of Chile doctoral grant number 21181027. VM was supported by ANID doctoral grant number 21200394 and CONACyT (Mexico) doctoral grant 739582. This article was financed by FIA PYT-2020-0462 project “Generacioín de plantas de vides de vino doble-resistentes para el fitopatoígeno *Erysiphe necator*, con buenas caracteriísticas enoloígicas’’ from the government of Chile, project FEQUIP2018-CI-04 and Corfo 13CTI-18862.

## Conflict of interest

Author PA-J was employed by the company Agrijohnson Ltda.

The remaining authors declare that the research was conducted in the absence of any commercial or financial relationships that could be construed as a potential conflict of interest.​

## Publisher’s note

All claims expressed in this article are solely those of the authors and do not necessarily represent those of their affiliated organizations, or those of the publisher, the editors and the reviewers. Any product that may be evaluated in this article, or claim that may be made by its manufacturer, is not guaranteed or endorsed by the publisher.
