# Supplementary material for: Characterization of physiological and antioxidant responses in Run1Ren1 Vitis vinifera plants during Erysiphe necator attack
Source: Front Plant Sci. 2022 Oct 6;13:964732. doi: 10.3389/fpls.2022.964732 (PMC9621084; doi:10.3389/fpls.2022.964732)
Supplement: Supplementary file 1 [file Presentation_1.pptx]

## Slide 1
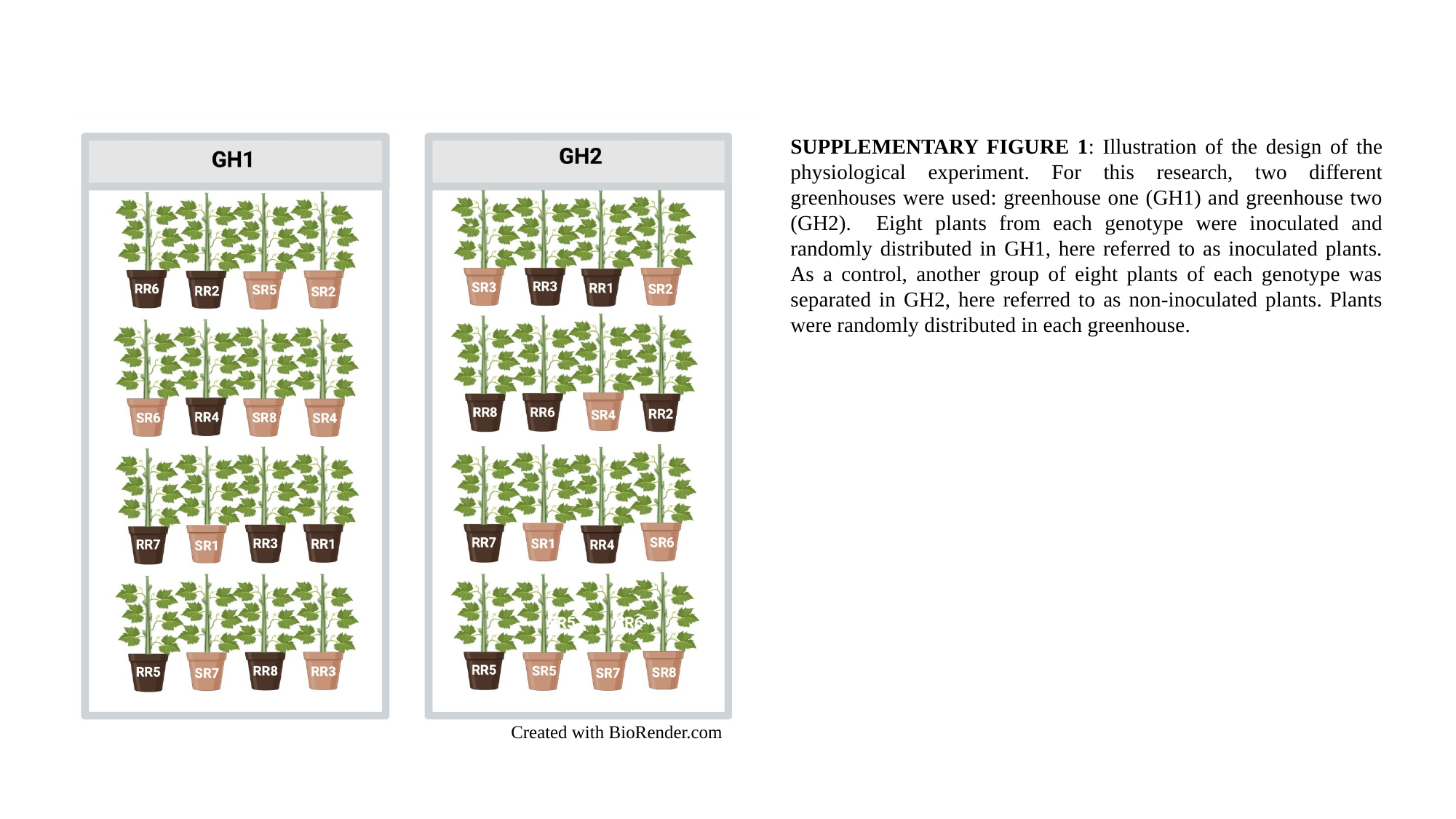

SUPPLEMENTARY FIGURE 1: Illustration of the design of the physiological experiment. For this research, two different greenhouses were used: greenhouse one (GH1) and greenhouse two (GH2). Eight plants from each genotype were inoculated and randomly distributed in GH1, here referred to as inoculated plants. As a control, another group of eight plants of each genotype was separated in GH2, here referred to as non-inoculated plants. Plants were randomly distributed in each greenhouse.
Created with BioRender.com
